# Platelet-Rich Concentrates in the Management of Lichen Planus—A Comprehensive Review

**DOI:** 10.3390/jcm14155368

**Published:** 2025-07-29

**Authors:** Zuzanna Ślebioda, Hélène Rangé, Agnieszka Mania-Końsko, Marzena Liliana Wyganowska

**Affiliations:** 1 Department of Periodontology and Oral Mucosa Diseases, Poznan University of Medical Sciences, 60-812 Poznan, Poland; agnieszkamania@ump.edu.pl (A.M.-K.); klchstomiper@ump.edu.pl (M.L.W.); 2 Department of Periodontology, Faculty of Odontology, CHU Rennes, University of Rennes, 35000 Rennes, France; helene.range@univ-rennes.fr; 3 CIC 1414 (Centre d’Investigation Clinique de Rennes), CHU Rennes, University of Rennes, Inserm, 35000 Rennes, France; 4 UMR S 1317 Institut Numecan (Nutrition, Métabolismes et Cancer), 35000 Rennes, France

**Keywords:** oral lichen planus, oral pathology, periodontology, platelet-rich fibrin, platelet-rich plasma

## Abstract

**Background:** Oral lichen planus is a chronic, potentially malignant disorder affecting the mucous membrane. As the etiology remains not fully understood, the treatment of this condition is mainly symptomatic, involving corticosteroids and other immunosuppressive agents, e.g., calcineurin inhibitors. One of the alternative therapeutic approaches includes platelet concentrates, which are autologous bioactive materials. The aim of this review was to evaluate the effects of platelet concentrates in the treatment of oral lichen planus and to compare them to other therapeutic strategies. **Methods:** The electronic databases PubMed/Medline, Web of Science, and Cochrane Library were searched for articles published up to 30 March 2025, describing clinical studies focused on oral lichen planus and treatment with platelet concentrates. **Results:** Fourteen studies describing the effects of oral lichen planus therapy with three types of platelet concentrates (injectable platelet-rich plasma, injectable platelet-rich fibrin, and platelet-rich plasma gel) were included in this review. Comparative strategies included steroids and immunosuppressive agents. The treatment duration ranged from 3 weeks to 2 months. The follow-up period varied from 4 weeks to 6 months. In most of the studies, comparable efficacy was achieved for platelet derivatives and alternative treatments. Two of the studies demonstrated more beneficial effects for platelet concentrates compared to controls, while in one of the studies, more severe adverse reactions were revealed in the platelet group compared to the controls. **Conclusions:** Autologous platelet concentrates showed comparable efficacy in achieving clinical improvement in patients with oral lichen planus to steroids and immunosuppressive drugs. Platelet derivatives could be considered as an alternative treatment to topical immunosuppressives, especially in steroid-refractory cases.

## 1. Introduction

Lichen planus is a chronic inflammatory condition affecting skin and mucosal membranes, frequently with oral involvement only, named oral lichen planus (OLP) [1]. It develops due to a disturbed immunological response triggered by several endo- and exogenous factors, although the etiology has not been clearly established [1,2,3,4]. It is considered to be a T-cell-mediated condition that results from an autoimmune response of a cytotoxic type. It becomes subsequently aggravated by the cluster of differentiation 4+ (CD4+) T-cell lymphocytes, which infiltrate the lesions [4,5]. Meanwhile, cytotoxic CD8+ T-cell lymphocytes are involved in recognizing and attacking basal keratinocytes, which leads to the formation of characteristic lichenoid lesions. Several precipitating factors initiating the dysregulation of the immune system in OLP have been proposed, including genetic predisposition, autoimmune reaction, and systemic diseases (namely, diabetes, hypertension, and malignant neoplasms). Other potential enhancers involve infectious agents, stress, food allergies, reactions to drugs, and dental materials [2,3,4,5]. It presents with reticular, atrophic, erosive, or ulcerative lesions, which appear in a recurrent mode [2,3]. It often causes unpleasant symptoms, like pain and discomfort. It may lead to functional limitations, influencing the patients’ quality of life [3,4,5].

The prevalence of OLP in the general adult population reaches 2%, with the average age of onset between 30 and 60 years, and with a female predilection [3,4]. OLP in children is infrequent [3]. OLP was classified by the WHO as an oral potentially malignant disorder with a transformation risk from 0.4% to 3.3% over 0.5 to >20 years [6].

The treatment of OLP is mainly symptomatic, but an effective causative strategy has yet to be established. Dental plaque control has shown benefits in clinical disease score and oral health quality of life outcomes in patients with OLP [7]. In addition, various treatment approaches have been used to manage OLP, including topical and systemic drugs, phototherapy, and laser therapy [2,3,8,9]. The conventional strategy includes various pharmacological agents, most commonly corticosteroids. Alternate options include calcineurin inhibitors, azathioprine, mycophenolate mofetil, retinoids, dapsone, and hydroxychloroquine [2,3,8,9]. Corticosteroid therapy in both local and systemic forms can produce severe adverse effects, including pain, bleeding, and ulceration. Other common complications comprise secondary infections, hypopigmentation, and allergic reactions [8,9,10]. The effectiveness of steroid treatments varies. Therefore, alternative therapies for OLP are being constantly explored.

Autologous platelet concentrates (APCs) derived from the patient’s blood are used in oral surgery, implantology, and periodontology due to the ability to promote tissue healing and regeneration. Additional clinical benefits from APC treatment, adjunct to non-surgical and surgical periodontal therapy, were reported in several studies [11,12,13]. It has been considered an encouraging option for reducing post-extraction pain and improving tissue healing during alveolar ridge preservation (ARP) [12]. It is also believed that the adjunctive use of autologous platelet concentrates to periodontal surgical procedures produces a better and more predictable outcome for the treatment of infrabony defects [13]. APCs frequently utilized in dental treatment include platelet-rich plasma (PRP), platelet-rich fibrin (PRF), and platelet-rich growth factor (PRGF). Two other variants include platelet-rich fibrin matrix (PRFM), with a capacity to release the growth factors, and platelet-poor plasma (PPP), which is produced by further centrifugation to adjust the profile of growth factors [14,15]. The qualification system for PCs proposed by Dohan et al. in 2009 [16] was based on the method of preparation, differences in content, and properties of the resulting fibrin network. In this classification, four types were distinguished: pure PRP/leukocyte-poor PRP (PPP/P-PRP); leucocyte and PRP (L-PRP); pure PRF/leucocyte-poor PRF (P-PRF); and leucocyte and PRF (L-PRF) [17]. Depending on the protocol used, PRP is obtained by a single or double centrifugation of the patient’s whole blood and contains platelet concentrations that exceed physiological values, at least 2–3 times above normal [18,19]. The final formulation is affected by the time and speed of centrifugation [16,20].

Platelets are the carriers of numerous growth factors and signaling proteins, all of which have a key role in tissue repair as well as regeneration. Small amounts of leukocytes, which are responsible for the inflammatory response and immune processes, are also present in PRP, together with alpha granule components that include platelet-derived growth factor (PDGF), vascular endothelial growth factor (VEGF), epithelial growth factor (EGF), transforming growth factor beta (TGF-b), insulin-like growth factor (IGF), serotonin, dopamine, histamine, adenosine, and calcium. The release of all of these factors contributes to accelerated healing and better tissue regeneration [21,22,23]. PRF is a biological preparation full of platelets and growth factors. This three-dimensional fibrin network plays a pivotal role in the modulation of the immune response and neovascularization [14]. PRF contains leukocytes and promotes wound healing by stimulating angiogenesis, immune regulation, and epithelial proliferation [14].

Despite promising results, the efficacy of PCs has not been clearly confirmed. The lack of a universal classification of PRP hinders the comparisons of the clinical trials’ results. There is a large diversity in the standardization of preparation methods, the differences in platelet concentration, and the presence of leukocytes. The effectiveness of PRP itself may depend on the patient’s age, health status, treatment time, medications taken, and number of administrations [24,25].

The aim of the present review is to summarize the literature reports on the safety and efficacy of PCs in the treatment of OLP and to compare them with other treatment approaches.

## 2. Search Strategy and Study Selection

The population consisted of human individuals affected by OLP, diagnosed through clinical evaluation or histopathological examination. The analyzed intervention was the use of injectable PRF (i-PRF), injectable PRP (i-PRP), and PRP gel. The comparison included other therapeutic approaches, such as corticosteroids and pharmaceutical interventions, or a placebo. Outcomes of interest were related to the efficacy of pain reduction and improvement in lesion size as primary outcomes, and remission time, blood immune indices, and salivary interleukin levels as secondary outcomes. Safety outcomes were also collected. The study design included randomized clinical trials, cohort studies (prospective or retrospective), case–control studies, and case reports.

The electronic databases PubMed/Medline, Web of Science, and Cochrane Library were searched for articles published up to 30 March 2025, with different Medical Subject Headings (MeSH) and supplementary non-MeSH terms, including “oral lichen planus” and “platelet-rich fibrin” or “platelet-rich plasma” or “platelet-rich concentrates”. The full-text articles in English were qualified for further evaluation. A manual search of the potentially eligible references was also performed. Initially, the records were assessed for the relevance of the title and/or abstract. Further analysis of the full texts included the studies that met the following inclusion criteria: human clinical studies analyzing the efficacy of PRP or PRF in OLP, or comparing these treatments to standard care. The reviewers were not blinded to the authorship of the studies. The extracted data included authorship, year of publication, country of origin, type of intervention and comparisons, study population and design, follow-up period, and outcomes.

## 3. Results

### 3.1. Study Characteristics

We analyzed fourteen studies that evaluated the safety and efficacy of PCs in the treatment of OLP and/or compared them with other treatment approaches or a placebo: four from Egypt, four from India, two from Italy, and one each from Australia, China, Iraq, and Türkiye. Treatments included i-PRF [26,27,28], i-PRP [29,30,31,32,33,34,35,36,37,38], and PRP gel [39]. Treatment duration ranged from 3 weeks [36], 4 weeks [26,27,30,32,35,37,38,39], 1 month [31], 6 weeks [34], 8 weeks [28], and 2 months [29,33]. The follow-up periods varied: 4 weeks [27,36], 2 months [28,34,35,38], 3 months [26,32,33], 4 months [29,31], 24 weeks [26], and 6 months [37]. In one study, there was no follow-up [39]. The efficacy outcomes were assessed using a visual analog scale (VAS) (nine studies; [26,27,28,29,30,31,34,37,39]), the score of reticulation/keratosis, erythema, and ulceration (REU) (two studies; [26,33]), the Thongprasom score (two studies; [27,32]), the OHIP-14 score (one study; [28]), the erythema scale (one study; [29]), the numeric rating scale (NRS) (one study; [33]), Modified Escudier Index-10 (a clinical disease status) (one study; [35]), and objective signs score (one study; [39]), and by evaluating changes in lesion diameter (seven studies; [26,28,29,30,33,34,37]), lesion signs and symptoms (three studies; [33,36,38]), signs of inflammation (one study; [37]), remission time (one study; [32]), blood immune indices (CD3+, CD4+ T cells and CD4+/CD8+ ratios) (one study; [39]), and changes in salivary TNF-α (one study; [35]) and IL-8 (one study; [37]). The safety outcomes were evaluated with reported side effects. Five of the described studies were split-mouth RCTs [26,27,28,31,35], two were cohort studies [30,36], and two were case reports [38,39]. Two other studies were case–control studies [29,33]. The remaining three were RCTs [32,34,39].

Table 1 shows the main characteristics of the studies included in this review and the most essential conclusions.

### 3.2. Results of the Studies

As presented in Table 1, three treatment modalities utilizing PCs were analyzed in this review, namely, i-PRF, i-PRP, and PRP gel. In most of the studies, the treatment efficacy in OLP patients was compared with steroidal therapy, i.e., triamcinolone acetonide (TA) or methylprednisolone acetate (MA), while in one study it was compared with tacrolimus, and in another study, with mycophenolate mofetil (MMF). As there was some heterogeneity in the study design, treatment strategy, and the outcome assessment measures, some conclusions can be stated, although they need to be treated with caution.

In all three studies that evaluated the efficacy of i-PRF, a comparative arm utilized steroids (in two studies, it was triamcinolone acetonide [26,27], while in one study, it was methylprednisolone acetate [28]). Studies were designed as split-mouth RCTs. The conclusions were consistent for all three studies and showed clinical improvement in all study participants at the end of the follow-up period, which ranged from 4 to 24 weeks, with no statistically significant differences between the groups. The value of the OHIP-14 index, reflecting the patient’s quality of life and assessed in the study by Saglam et al., increased in both compared groups in a similar way [28].

Injectable PRP was the treatment strategy evaluated in the ten subsequent studies included in this review [29,30,31,32,33,34,35,36,37,38]. In six studies in a comparative arm, TA was used. In a study by El Araby et al., an additional treatment option apart from i-PRP and TA included MMF [34]. In most of the studies, the clinical outcomes were comparable for the tested treatment strategies [29,31,32,33]. However, as observed by ElGhareeb et al. [33], although statistically significant decreases in REU and pain scores were found in both groups after treatment compared to before, the frequency of side effects among patients who received PRP was higher than in the TA group. The recurrence rate during the 3-month follow-up was also more significant among patients treated by PRP [33]. In the studies by Choudhary et al. and El Araby et al., i-PRP showed more beneficial effects in terms of clinical presentation compared to TA [34,35]. Two other studies were designed as a cohort observation with no comparative therapy [30,36], and the other two studies were case reports, also without controls [37,38]. In a cohort study by EL-Shinnawi et al., i-PRP showed significant efficacy in ameliorating the signs and symptoms in 10 steroid-resistant erosive OLP cases [30]. In a cohort study by Asal et al. [36], the majority of OLP patients showed an increased salivary IL-8 level after PRP, though the difference between the baseline and follow-up period was statistically insignificant. I-PRP relieved the signs and symptoms of OLP lesions and turned hyperemic lesions into a normal mucosal color, but the dimensions of the lesions were resistant to change [36]. Significant reduction in patient symptoms and complete regression of the lesion after 2 months was described in Merigo et al.’s case report, similarly to the case presented by Shaik et al., who observed a reduction in patient symptoms and an improvement in clinical presentation of the lesion after one week, with complete regression of the lesion after the fourth week [37,38].

The efficacy of PRP gel versus tacrolimus was evaluated in Huang and Li’s study. They demonstrated significantly reduced lesion area scores and VAS scores compared to baseline in both groups. However, the study group showed statistically significantly lower scores than the controls. During follow-up, the percentages of CD3+, CD4+ T cells, and CD4+/CD8+ ratios in the PRP group were greater than those in the control group (differences were statistically significant) [39].

## 4. Discussion

The therapeutic application of autologous PCs represents a relatively new biotechnology that has become a breakthrough in stimulating and accelerating soft tissue and bone healing. Its effectiveness lies in the local and continuous delivery of a wide range of growth factors and proteins, mimicking the needs of physiological wound healing and tissue repair processes. The idea of using PRP was pioneered in the 1970s, in the field of hematology, to treat patients with thrombocytopenia. In the 1980s and 1990s, platelet-rich plasma was used in surgical procedures, mainly in plastic and maxillofacial surgery [17]. Nowadays, it is widely used in several fields of medicine and dentistry due to its beneficial effects. It accelerates healing after tooth extractions, supports bone augmentation, promotes ligament and cartilage regeneration, aids the treatment of androgenetic alopecia, and reduces acne scars, swelling, and bruising after surgery [20,40,41,42,43,44].

For these reasons, the utilization of APCs in the treatment of OLP seems to be justified. OLP is often resistant to therapy, with common recurrences, even though various treatment approaches have been proposed [2,3,9]. The treatment strategy in this disease is mainly symptomatic and often involves immunosuppressive agents. Even if used topically, they may generate several side effects, especially if applied in a repeated manner. Short-term use of steroids may result in increased appetite, weight gain with unusual fat distribution around the abdomen or across the upper back, insomnia, depression, euphoria, and confusion. The adverse effects of the long-term use of steroids include high blood pressure, high blood sugar, osteoporosis and fractures, thinning of the skin, bruising, poor wound healing, and muscle weakness. Life-threatening complications of steroidal treatment involve adrenal crisis and severe immunosuppression [14]. Another potential complication of this treatment strategy is topical corticosteroid withdrawal (TSW), an entity associated with chronic steroid use and misuse [10]. Although immunosuppressive agents reduce the severity and progression of OLP, they may conjointly trigger malignant transformation. Therefore, there is an urgent need to establish alternative treatment strategies for OLP patients that are potentially safer and more effective.

In this review, it was shown that platelet-rich concentrates are an acceptable strategy in the management of OLP with a comparable efficacy to steroids and other immunosuppressives (tacrolimus, MMF). Injectable PRF presented as an effective treatment strategy in three RCTs included in this review, with no statistically significant differences between the study group and controls treated with steroids [26,27,28].

In six studies comparing i-PRP and TA, the clinical outcomes were also beneficial and comparable for the tested treatment strategies [31,32,33,34]. In two of those studies, i-PRP presented even more advantageous therapeutic effects in terms of clinical presentation compared to TA [34,35]. However, in a study by ElGhareeb et al., the frequency of side effects among patients who received PRP was higher than in the TA group, while the recurrence rate during the 3-month follow-up was also higher among patients treated by PRP [33]. The authors reported that the pain frequency was higher among patients who received PRP compared to intralesional corticosteroids. This, however, could be justified by the dilution of corticosteroids with a local anesthetic, lidocaine, which reduced the discomfort in the steroid arm of the study [45]. The differences in the frequency of side effects between the study groups were only reported in this one study included in our review; therefore, any conclusions need to be stated with caution. However, in light of the previously emphasized need to establish a treatment strategy for OLP that is safer than steroids, it should draw attention to the need for a more extensive evaluation of APCs’ safety. Until now, this therapeutic approach was considered nearly free from adverse effects [41,42,43,44]. However, as the popularity of this treatment modality has constantly been increasing, there is an urgent need for future safety-focused studies.

It is worth noting that i-PRP showed significant efficacy in ameliorating the signs and symptoms in steroid-resistant erosive OLP cases. Based on EL-Shinnawi et al.’s [30] observations, i-PRP was shown to be effective in decreasing the symptoms and improving the clinical signs of OLP that do not respond to conventional therapy. In their study on a selected group of 10 patients with recurrent erosive OLP that was unresponsive to previously used forms of treatment, the complete remission of oral discomfort and improvement in the quality of life indices were achieved. There were no reported relapses or recurrences of the lesion after cessation of therapy up to three months, and no adverse effects of i-PRP were observed [30].

**Figure 1:**
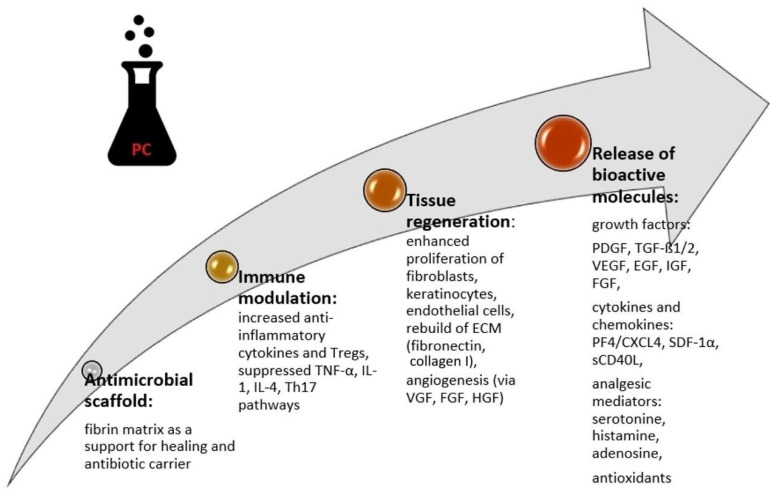
Biologic actions of platelet concentrates.

In the study where MMF was used as an additional control, the treatment efficacy was also comparable in all study groups [34]. High treatment efficacy in OLP, similar to topical tacrolimus, was demonstrated in Huang et al.’s [39] study of PRP in gel. The comparable effects of PCs and steroids can be explained by the anti-inflammatory potential of both of these therapeutic strategies. Corticosteroids stimulate an anti-inflammatory response in oral keratinocytes and present the ability to locally regulate T-lymphocytes [46]. Meanwhile, PRF inhibits the action of TLR4+ cells, which are primarily expressed in macrophages, dendritic cells, and some epithelial cells. These cells recognize lipopolysaccharides. In this way, it also promotes and triggers an anti-inflammatory response. PRF reduces the activation of TLR4 and blocks the classical inflammatory-related signaling cascade, mainly via the inhibition of p-p65. For these anti-inflammatory properties, PRF may serve as an effective alternative therapeutic approach in OLP [42]. The biologic actions of PCs are presented in Figure 1.

Our observations stand in line with the conclusions derived in a recent systematic review by Niemczyk et al., who stated that PRP and i-PRF represented biocompatible and less invasive treatment alternatives with a reduced incidence of adverse effects compared with conventional therapeutic approaches for OLP [46]. Also, based on Gupta et al.’s [15] meta-analysis and systematic review, i-PRF seems to be an encouraging strategy in the management of pain and burning sensation, reducing lesion size, and enhancing patient satisfaction in OLP. The authors demonstrated the potential significance of i-PRF in managing OLP [15].

One of the aspects potentially hindering the wide access to therapy with PCs in OLP is the cost, which is higher than in steroid therapy. In Sandhu et al.’s systematic review, based on the estimated cost per month and evidence for efficacy and side effects in OLP, topical steroids appeared to be more cost-effective than topical calcineurin inhibitors, followed by intra-lesional triamcinolone [47]. The authors did not include PCs as a treatment option in this comparison; nevertheless, currently, this strategy may not be widely accessible for this reason. The broad use of this treatment approach may also be influenced by geographic variability in access. North American and Chinese PRP research centers are very advanced, though the treatment in the USA is considered relatively expensive. European countries such as Germany, Spain, and Switzerland have been at the forefront of PRP innovations, though the regulations vary across countries, which may limit accessibility for patients. Türkiye, Thailand, and South Korea are well-recognized PC therapy providers in cosmetic dermatology and hair restoration [48,49].

Based on this review, PRF and PRP present as promising new lines of treatment in OLP. Future multi-center RCTs with larger sample sizes are required to establish more detailed therapeutic algorithms and to evaluate the treatment safety, which would also consider the prevalence, incidence, and baseline severity of OLP in populations from different world regions.

## 5. Limitations

This review was based on a relatively small sample size of studies, which may interfere with the validity. Moreover, a certain heterogeneity of the methods used in the included studies was found; there was a variety of doses, number of sessions, and duration of follow-up performed. In all studies, the efficacy of the treatment modalities was only assessed using qualitative scales (VAS, REU, or OHIP-14). The lack of histopathological evaluation undermines the evaluation of the study results. Lastly, the adverse effects of PCs and other treatments were rarely reported in the included studies. There is an urgent need for future studies on this topic to include histopathological endpoints and to use standardized outcome measures.

## 6. Conclusions

Autologous platelet concentrates showed comparable efficacy in achieving clinical improvement in patients with oral lichen planus to steroids and immunosuppressive drugs.

Platelet derivatives could be considered as an alternative treatment to topical immunosuppressives, especially in steroid-refractory cases. PRCs may offer a steroid-sparing option in selected patients.

## Figures and Tables

**Table 1:** Characteristics and main results of the studies included in the review.

Author, Year, Country	Study/ Control Group (n)	Intervention/ Comparison	Study Design	Treatment Duration/Follow-up Period	Efficacy Outcome Assessment/Side Effects	Results
Al-Hallak et al., 2023, Australia [26]	12 (12/12)	i-PRF TA	Split-mouth RCT	4 weeks/3 months	VAS, REU, lesion diameter	The average VAS and REU scores decreased with no statistically significant differences between the groups
Bennardo et al., 2021, Italy [27]	9 (9/9)	i-PRF TA	Split-mouth RCT	4 weeks/4 weeks	VAS, Thongprasom score	The average VAS and Thongprasom scores decreased with no statistically significant differences between the groups at the end of the follow-up
Saglam et al., 2021, Türkiye [28]	24 (24/24)	i-PRF MPA	Split-mouth RCT	8 weeks/24 weeks	VAS, OHIP-14, lesion diameter	A reduction in pain and lesion size and an increase in satisfaction was observed in both groups
Ahuja et al., 2020, India [29]	20 (10/10)	i-PRP TA	Prospective case–control RCT	2 months/4 months	VAS, erythema scale, lesion diameter	The efficacy of i-PRP was similar to i-TA in erosive OLP. PRP therapy exhibited less recurrence and no adverse effects
Hijazi et al., 2022, Egypt [32]	20 (10/10)	i-PRP TA	Pilot RCT	4 weeks/3 months	NRS, Thongprasom score, remission time	Both groups showed significant improvement in the clinical parameters, with no statistical significance when comparing the two groups in pain score, clinical score, or remission
ElGhareeb et al., 2023 Egypt [33]	24 (12/12)	i-PRP TA	Case-control	2 months/3 months	REU, NRS, lesion diameter	Statistically significant decrease in REU and pain score in both groups after treatment compared to before; the frequency of side effects among patients who received PRP was higher than in the TA group. The recurrence rate during the 3-month follow-up was more significant among patients treated by PRP
Choudhary et al., 2023, India [35]	28 (28/28)	i-PRP TA	Split-mouth RCT	4 weeks/2 months	Modified Escuider Index-10	A statistically significant reduction in the lesion activity scores was found amongst i-PRP group as compared to the TA group
Sharma et al., 2023, India [31]	20 (20/20)	i-PRP TA	Split-mouth RCT	1 month/4 months	VAS, Lesion signs and symptoms	The effectiveness of i-PRP and TA in reducing the size of the lesion was comparable. I-PRP showed slightly better results in reducing the severity of the lesion and in pain and burning
El Araby et al., 2025, Egypt [34]	30 (10/10/10)	i-PRP MMF TA	RCT	6 weeks/2 months	VAS, lesion diameter salivary TNF-α	At 2, 4, 6, and 8 weeks, the comparison of clinical parameters was in favor of i-PRP with significant reduction in pain and size of lesion and with marked decrease in TNF-α amount in saliva
Asal et al., 2023, Iraq [36]	13 (13/0)	i-PRP	Cohort	3 weeks/4 weeks	Lesion signs and symptoms, salivary IL-8	Insignificantly increased salivary IL-8 after i-PRP; i-PRP relieved OLP lesions’ signs and symptoms, and turned hyperemic lesions into normal colour, but lesions’ dimensions were resistant to change
EL-Shinnawi et al., 2021, Egypt [30]	10 (10/0)	i-PRP	Cohort	4 weeks/2 months	VAS, lesion diameter	i-PRP showed significant efficacy in ameliorating the signs and symptoms in steroid-resistant erosive OLP cases
Sriram et al., 2020, India [37]	1	i-PRP	Case report	4 weeks/6 months	VAS, lesion diameter, signs of inflammation	Significant reduction in patient symptoms and clinical signs after one week, and complete regression of the lesion after the 4th week
Merigo et al., 2018, Italy [38]	1	i-PRP	Case report	4 weeks/2 months	Lesion signs and symptoms	Significant reduction in patient symptoms and complete regression of the lesion after 2 months
Huang and Li, 2024, China [39]	80 (40/40)	PRP gel Tacrolimus gel	RCT	4 weeks/No follow-up	Objective signs score, VAS, blood immune indices (CD3+, CD4+ T cells, and CD4+/CD8+ ratios)	Significantly reduced lesion area scores and VAS scores compared to baseline in both groups, with the study group showing statistically significantly lower scores than the control group. The percentages of CD3+, CD4+ T cells, and CD4+/CD8+ ratios in the study group were greater than those in the control group (differences statistically significant) in the post-treatment period

i-PRF: injectable platelet-rich fibrin; TA: triamcinolone acetonide; RCT: randomized controlled trial; MPA: methylprednisolone acetate; VAS: visual analog scale; REU: reticular/hyperkeratotic, erosive/erythematous, ulcerative; OLP: oral lichen planus; OHIP-14: Oral Health Impact Profile-14; NRS: numerical rating scale; MMF: mycophenolate mofetil; IL-8: interleukin-8.

## Data Availability

The datasets analyzed during the current study are available in the cited publications and can be provided by the corresponding author upon request.
